# Evaluation of Two Commercial DNA-Based Breed Identification Tests for Pit Bull-Type Dogs in Taiwan: A Pilot Study

**DOI:** 10.3390/ani16182896

**Published:** 2026-09-15

**Authors:** Yung-Yun Yang, Ann Nee Lee, Tzu-Chieh Chen, Wei-Hsiang Huang

**Affiliations:** Graduate Institute of Molecular and Comparative Pathobiology, School of Veterinary Medicine, National Taiwan University, Taipei 10617, Taiwan; f13644002@ntu.edu.tw (Y.-Y.Y.); r09644004@ntu.edu.tw (A.N.L.); tzucchen@ntu.edu.tw (T.-C.C.)

**Keywords:** pit bull-type dogs, breed identification, visual breed identification, DNA breed identification, single nucleotide polymorphism, breed-specific legislation

## Abstract

The “Declaration of Specified Breed Names of Animals Banned from Being Owned, Imported, or Exported” was promulgated in Taiwan in response to aggression involving pit bull-type dogs. To support enforcement, we developed standards for visual breed identification based on international breed standards and UK guidance. Given the subjectivity of visual identification, samples from 21 healthy dogs, including 12 pit bull-type dogs, were submitted to Embark^®^ and Orivet Genetic Pet Care for SNP-based breed identification. Across pit bull-type dogs, American Pit Bull Terrier signatures reported by Embark^®^ were reported as American Staffordshire Terrier by Orivet, while Formosan Mountain Dog signatures were reported as mixed breeds. Embark^®^ covered all four pit bull-type breeds and the Formosan Mountain Dog and produced fewer failed tests, providing broader coverage of breeds relevant to Taiwan. However, substantial discordance was observed between the two services in the assignment of the American Pit Bull Terrier and American Staffordshire Terrier, the two breeds specified in the Taiwanese declaration. This findings suggest that commercial DNA breed identification requires further validation before it can support regulatory enforcement.

## Simple Summary

Pit bull-type dogs are regulated in Taiwan, but identifying these dogs by appearance alone can be subjective and unreliable. We compared visual breed identification with two commercial DNA testing services using 21 dogs, including 12 pit bull-type dogs. The two DNA services sometimes assigned different breed names to the same dogs, particularly for the American Pit Bull Terrier and American Staffordshire Terrier. Embark^®^ provided broader coverage of breeds relevant to Taiwan and had fewer failed tests. However, the two services showed substantial discordance in breed assignments among closely related pit bull-type breeds, particularly the American Pit Bull Terrier and American Staffordshire Terrier. Commercial DNA testing may therefore provide supplementary information for breed identification, but validation using dogs of independently verified ancestry would be required before regulatory use.

## 1. Introduction

Aggression of pit bull-type dogs causing traumatic accidents involving dogs and humans has been a topic of worldwide discussion for decades. Recently, there has been an increase in biting accidents reportedly caused by pit bull-type dogs, reaching up to six cases compared with the two to three cases in previous years in Taiwan. Although owners were fined under Article 20 of the Animal Protection Law, arguments remain regarding banning pit bull-type dogs.

On 26 October 2021, the Council of Agriculture, Executive Yuan, Republic of China amended the “Declaration of Specified Breed Names of Animals Banned from Being Owned, Imported, or Exported” under Article 8 and Article 36 of the Animal Protection Law, which states that the American Pit Bull Terrier and the American Staffordshire Terrier are banned from being owned, imported, or exported. “Pit bull” is not a recognized breed but a heterogeneous group that may include purebred dogs of various breeds and dogs presumed to be mixes of those breeds [1]. Nor is “Pit Bull Terrier” a classification of breeds, as “Staffordshire Bull Terrier,” “American Staffordshire Terrier,” “American Bulldog,” and “Bull Terrier” are each accepted individually by the Fédération Cynologique Internationale (FCI) under the “Bull Type Terriers” heading [2]. Therefore, in this study, the term “pit bull-type breeds” is used to include American Pit Bull Terriers, American Staffordshire Terriers, Staffordshire Bull Terriers, American Bullies, and their mixes, because the term is frequently used in laws regulating dog ownership based on breed or phenotype [1].

Breed standards based on kennel-club pedigree registries or visual identification can be confusing. This is because individual breeds are variably recognized by the respective worldwide or widely recognized kennel-club pedigree registries, namely the Fédération Cynologique Internationale (FCI), American Kennel Club (AKC), The Kennel Club (tKC), and United Kennel Club (UKC). Both the FCI and AKC recognize only the Staffordshire Bull Terrier and the American Staffordshire Terrier; the tKC recognizes only the Staffordshire Bull Terrier; and the UKC recognizes the American Pit Bull Terrier, Staffordshire Bull Terrier, and American Bully [2,3,4,5].

However, visual breed identification is often inaccurate. Experts, including individuals from veterinary medical groups, animal control or sheltering agencies, dog clubs, and regional and national conferences, have very little agreement when determining breeds visually [1,6,7,8]. In one study, the sensitivity and specificity of visual breed identification were found to fall within a wide range, with poor agreement between observers [1]. Although the shelter staff in one study achieved 67.7% breed matches, this fell to 10% for primary and secondary breed matches combined [6].

Over the decades, legislation related to dog aggression has been implemented in various countries and categorized into two types: dangerous dog laws and breed-specific legislation (BSL). BSL includes laws that prohibit or restrict particular breeds or types of dogs, whereas dangerous dog laws focus mainly on the “behavior” of dogs rather than the breed label. Different countries have incorporated these two categories of legislation to various degrees. BSL has been implemented in Ireland, Spain, and parts of the United States and Canada, whereas both types of law have been implemented in the United Kingdom [9,10,11,12]. Regarding BSL in several U.S. states during the 20th century, when a “pit bull breed” was defined as any dog exhibiting distinguishing characteristics that “substantially conform to the standards” of the American Kennel Club or United Kennel Club, some courts struck down such ordinances as vague, owing to the difficulty even for experts of determining the breeding of mixed-breed dogs [13].

In addition to mere visual breed identification, there are more objective measures such as DNA analysis. Molecular markers have been widely used for parentage, evolutionary relationships, and breed and species identification in domestic animals and wildlife over the past three decades. Single nucleotide polymorphisms (SNPs), short tandem repeats (STRs), and mitochondrial DNA (mtDNA) have been described in studies related to canine breed identification. Studies have revealed that mtDNA is poorly correlated with breeds [14,15,16,17,18]. As for STRs, several studies have used markers of differing numbers of loci and generated pairwise distance matrices using the neighbor-joining algorithm to form homogeneous groups within populations of different canine breeds [19,20].

A SNP is a single polymorphic nucleotide in a sequence; it is the most common genetic polymorphism and has been proven reliable for breed identification by several commercial companies, such as Wisdom Panel™, Embark^®^, and Orivet Genetic Pet Care. Different technologies and protocols are used, each with a large established database. SNP genotyping is currently the most widely adopted approach for canine breed ancestry inference, owing to its high throughput and relatively low per-sample cost. Wisdom Panel™ uses a custom SNP microarray interrogating approximately 100,000 genomic locations and currently screens for more than 430 breeds and populations, with ancestry tracing across three generations. Embark^®^ uses a custom high-density SNP microarray containing hundreds of thousands of probes and screens for more than 400 breeds, types, and varieties. Orivet Genetic Pet Care currently screens for more than 350 breeds, types, and varieties and provides a three-generation ancestry analysis. Therefore, the pilot study aimed to compare breed ancestry assignments generated by two commercial SNP-based dog breed identification services, with particular emphasis on pit bull-type dogs, and to evaluate the concordance and potential limitations of these tests for forensic and regulatory applications in Taiwan. In addition, breed standards relevant to pit bull-type dogs were reviewed to provide a practical reference for first-line visual identification by regulatory personnel.

## 2. Materials and Methods

### 2.1. Literature-Based Comparative Synthesis of Breed Standards for Pit Bull-Type Dogs

A literature-based comparative framework was developed to support first-line visual identification of pit bull-type dogs. Breed standards for American Pit Bull Terrier, American Staffordshire Terrier, Staffordshire Bull Terrier, and American Bully were retrieved from major kennel club registries, including the Fédération Cynologique Internationale (FCI), American Kennel Club (AKC), The Kennel Club (tKC), and United Kennel Club (UKC). Guidance for enforcers under the Dangerous Dogs Act 1991 issued by the United Kingdom Department for Environment, Food and Rural Affairs (DEFRA) was also reviewed. Morphological descriptors and breed-defining characteristics were extracted and compared across sources, and the key features were compiled into a reference table for practical first-line visual identification.

### 2.2. Selection of DNA Typing Institutions or Commercial Companies

Commercial companies providing canine breed identification services, including Wisdom Panel™ (Mars Petcare, Portland, OR, USA), Embark^®^ (Embark Veterinary, Inc., Boston, MA, USA), and Orivet Genetic Pet Care (Orivet Genetic Pet Care Pty Ltd., St. Kilda, VIC, Australia), were initially considered. Other commercial companies were excluded because their breed reference databases are primarily based on the American Kennel Club (AKC) standards, which do not recognize the American pit bull terrier as a single homogeneous breed.

Among the three companies evaluated, Wisdom Panel™ indicated that its regional databases are limited to the United States, Canada, the United Kingdom, and Germany, with no database available for other regions, including Taiwan. Based on communication with the company, the service was not recommended for samples originating from regions outside their database coverage. Therefore, Wisdom Panel™ was excluded from further analysis.

Consequently, Embark^®^ and Orivet Genetic Pet Care were selected for this study. Embark^®^ utilizes a SNP genotyping platform based on Illumina’s CanineHD SNP array, and only breeds contributing at least 5% of the genetic breed signature are reported in the final results. Orivet Genetic Pet Care also performs SNP-based breed identification using a proprietary reference database; however, details regarding the genotyping platform and number of markers used were not disclosed by the company. The advantages, limitations, and breed identification results generated by the two laboratories were subsequently compared.

### 2.3. Sample Collection and DNA Analysis

Dogs were recruited through collaboration with the Kennel Club of Taiwan (KCT) and the Hsinchu County Pet Trading Association. Owners of dogs participating in the 2020 KCT National Dog Headquarters Exhibition Competition were invited to participate in the study on a voluntary basis. Sampling kits containing detailed written instructions were provided to participating owners. Samples were collected by the owners following the provided protocol, with assistance from the research team when necessary. For dogs that did not attend the exhibition, sampling kits were mailed to their owners, who collected the samples themselves following the same protocol.

Each dog was photographed, and saliva or oral mucosal swab samples were collected for DNA analysis. A Genotek PG-100 sponge swab (DNA Genotek, Ottawa, ON, Canada) was used for the Embark^®^ protocol with a sampling duration of 30–60 s, whereas sterile nylon brushes were used for the Orivet Genetic Pet Care protocol with a sampling duration of approximately 10 s. The samples were shipped to Embark^®^ and Orivet Genetic Pet Care at room temperature following the sampling instructions provided by each company. DNA extraction and SNP-based breed identification were performed by the respective companies according to their protocols. The results were subsequently retrieved from the online platforms provided by the companies.

### 2.4. Definition of Agreement

Agreement between owner-reported (or KCT-approved) breed and a DNA breed signature was defined a priori as the owner-reported breed being reported as the primary (highest-percentage) breed in that signature. Inter-laboratory agreement was defined as both services reporting the same primary breed. Dogs for which either service returned a failed test, or for which the owner did not specify a breed (Individual 02, reported only as “pit bull-type dog”), were excluded from the corresponding agreement calculation. All agreement figures are descriptive; no accuracy estimates are reported because no independent gold standard was available.

### 2.5. Use of Generative Artificial Intelligence

Generative AI was used solely to assist with manuscript drafting and language refinement and was not used in study design, data collection, data analysis, or interpretation of the results.

## 3. Results

### 3.1. Establishment of First-Line Visual Breed Identification of Pit Bull-Type Dogs

The breed standards based on the FCI, AKC, tKC, and UKC are compiled in Table 1 [2,3,4,5]. American Pit Bull Terriers were longer but more slender, whereas American Staffordshire Terriers were shorter but heavier, with a stockier, bulkier body. The Staffordshire Bull Terrier was the smallest among the four pit bull types. The weight of the American Bully also varied depending on the standards set by different kennel clubs. The muzzle length of the American Pit Bull Terrier and the American Bully was shorter than the length of the skull, with a ratio of approximately 2:3—about 40% of the overall length of the head for the former and 25–35% for the latter. Wrinkle formation on the forehead was noted when the American Pit Bull Terrier was concentrating.

There were 15 guidelines in the Guidance for enforcers under the Dangerous Dogs Act 1991 issued by the Department for Environment, Food and Rural Affairs [21]. The standard used to identify a pit bull-type dog was based on the American Dog Breeders Association standard of conformation, as published in the Pit Bull Gazette, vol. 1, issue 3, 1977. The law did not require a suspected pit bull-type dog to fit the description perfectly; rather, a dog presenting a substantial number of the characteristics may be considered ‘more’ pit bull-type than any other type of dog.

### 3.2. Results of DNA Typing

**Figure 1:**
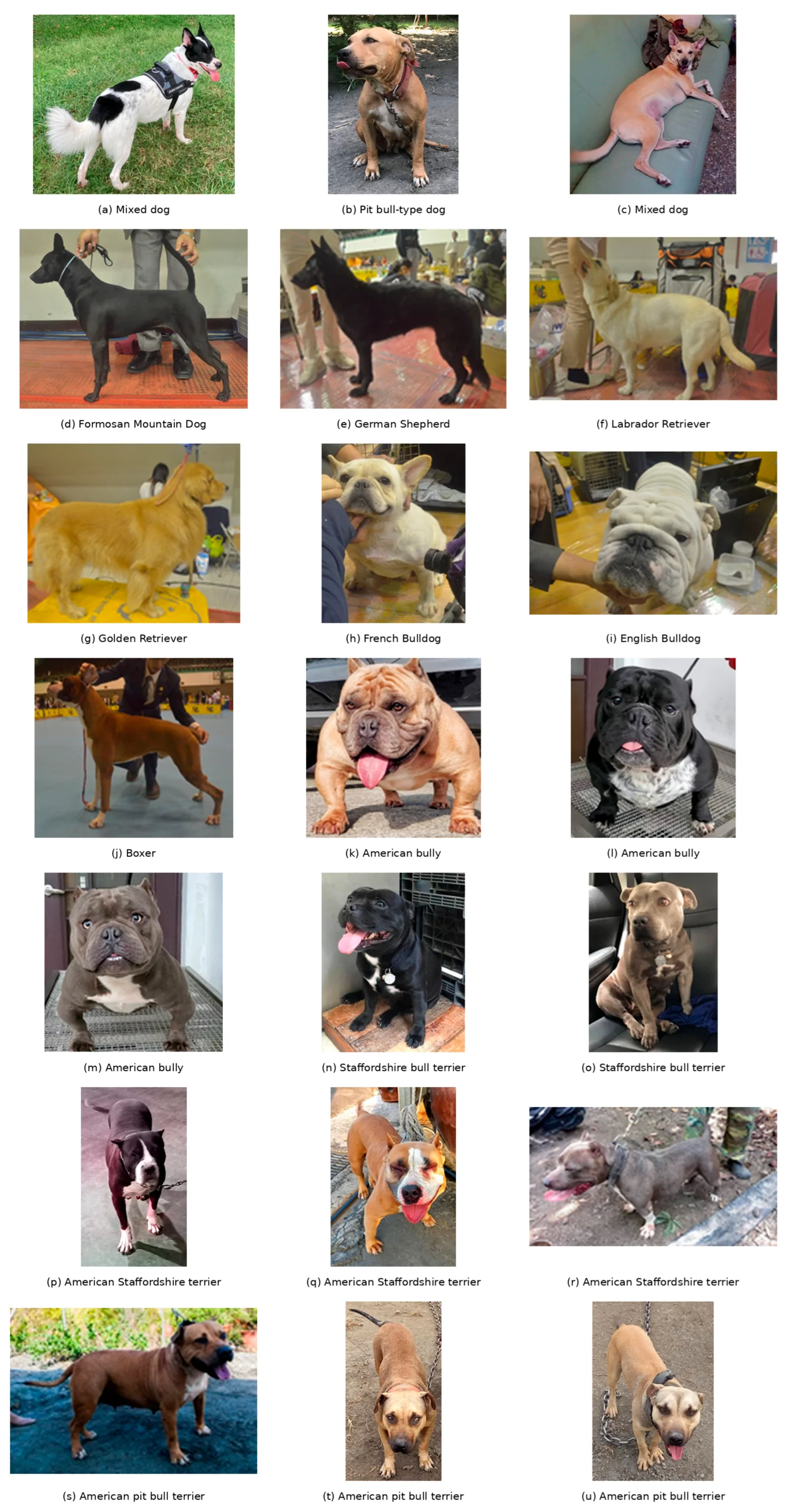
Individual dogs recruited in the current study. Breeds identified visually or approved by the Kennel Club of Taiwan are listed. (**a**) Individual 01: mixed dog; (**b**) Individual 02: pit bull-type dog; (**c**) Individual 03: mixed dog; (**d**) Individual 04: Formosan Mountain Dog; (**e**) Individual 05: German Shepherd; (**f**) Individual 06: Labrador Retriever; (**g**) Individual 07: Golden Retriever; (**h**) Individual 08: French Bulldog; (**i**) Individual 09: English Bulldog; (**j**) Individual 10: Boxer; (**k**) Individual 11: American Bully; (**l**) Individual 12: American Bully; (**m**) Individual 13: American Bully; (**n**) Individual 14: Staffordshire Bull Terrier; (**o**) Individual 15: Staffordshire Bull Terrier; (**p**) Individual 16: American Staffordshire Terrier; (**q**) Individual 17: American Staffordshire Terrier; (**r**) Individual 18: American Staffordshire Terrier; (**s**) Individual 19: American Pit Bull Terrier; (**t**) Individual 20: American Pit Bull Terrier; (**u**) Individual 21: American Pit Bull Terrier.

Twenty-one healthy dogs of various breeds were sampled by their respective owners. A total of 12 pit bull-type dogs were included, with breed identity visually determined by the owners and by breed consultants from KCT, including three American Pit Bull Terriers, three American Staffordshire Terriers, two Staffordshire Bull Terriers, three American Bullies, and one not specified. Meanwhile, nine dogs of other breeds were sampled as controls: two mixed-breed dogs, one Formosan Mountain Dog, one German Shepherd, one Labrador Retriever, one Golden Retriever, one French Bulldog, one English Bulldog, and one Boxer. Apart from the two mixed-breed dogs, the other control breeds were participants in the National Dog Headquarters Exhibition Competition 2020, and their breeds were approved by the KCT. Breeds of the pit bull-type dogs were provided by their respective owners. The 21 dogs are shown in Figure 1.

Embark^®^ and Orivet Genetic Pet Care were compared based on sampling method, sampling duration, sampling protocol, database, and other factors (Table 2). All four pit bull-type breeds are in the Embark^®^ breed database, whereas only the American Pit Bull Terrier, American Staffordshire Terrier, and Staffordshire Bull Terrier are in the Orivet Genetic Pet Care database. Moreover, Embark^®^ had advantages over Orivet Genetic Pet Care in several respects, including more than 230,000 SNP markers, a database of more than 400 breeds, a regional database for Taiwan, and downloadable raw data.

Failed tests occurred in 1/21 samples for Embark^®^ and 4/21 for Orivet Genetic Pet Care, although this difference was not statistically significant (Fisher’s exact test, *p* = 0.34). According to the reports provided by the commercial services, the failed tests were attributed to insufficient sample material and/or contamination. No sample-specific information regarding DNA quantity, DNA quality, contamination status, or other quality-control parameters was provided by either company.

Figure 1 shows the 21 dogs included in the study and their visually identified or KCT-approved breeds, whereas Table 3 summarizes the corresponding DNA breed identification results from Embark^®^ and Orivet Genetic Pet Care. Of the 21 samples, Embark^®^ returned results for 20 and Orivet Genetic Pet Care for 17, with both services returning results for 16 dogs.

Among the 12 pit bull-type dogs, four (Individuals 11, 16, 17, and 21) had a failed test in one laboratory and one (Individual 02) had no specific owner-reported breed; agreement with both laboratories could therefore be assessed in seven dogs, of which two (Individuals 14 and 15) agreed (2/7). Considering all 21 dogs, both services returned a result for 16, and the same primary breed was reported in 8 of these 16.

The six control purebred dogs (Individuals 05–10) were consistently assigned to their KCT-approved breeds by both laboratories. In contrast, recurring inter-platform discrepancies were observed for breeds relevant to the Taiwanese regulatory context. Formosan Mountain Dog ancestry reported by Embark^®^ was represented as complex mixed-breed ancestry by Orivet Genetic Pet Care, which does not include the Formosan Mountain Dog in its reference database. Similarly, dogs assigned American Bully ancestry by Embark^®^ were generally reported by Orivet Genetic Pet Care as American Staffordshire Terrier or combinations involving bulldog breeds.

A particularly consistent discrepancy involved the American Pit Bull Terrier and American Staffordshire Terrier. Orivet Genetic Pet Care did not report American Pit Bull Terrier ancestry in any dog, whereas dogs assigned American Pit Bull Terrier ancestry by Embark^®^ were assigned American Staffordshire Terrier ancestry by Orivet Genetic Pet Care, in some cases at identical ancestry proportions. Staffordshire Bull Terrier showed the greatest consistency, with Individuals 14 and 15 receiving concordant assignments from both laboratories. Overall, only 2 of the 7 evaluable pit bull-type dogs showed agreement between the owner-reported breed and both laboratory assignments.

## 4. Discussion

Based on the compilation of breed standards from different kennel clubs, nose and eye characteristics as well as coat color were included; however, these strict standards are usually used for scoring in dog shows and are thus not entirely relevant for first-line breed identification, because dogs that do not fit all of the described criteria cannot be excluded from the breed, whereas dogs that fit only some of the criteria are not necessarily mixes of pit bull-type dogs. 

A more practical approach to first-line breed identification for customs officers and government personnel is provided by the 15 guidelines of the United Kingdom Department for Environment, Food and Rural Affairs. These criteria include measurements and length ratios, which are more objective. The statement that a substantial number of characteristics should be met for a dog to be considered ‘more’ pit bull-type than any other type of dog is more practical to apply in reality. However, the same statement has been said to leave room for subjective interpretation [22].

Seven dogs in this study (Individuals 04–10) held pedigree certificates from the Kennel Club of Taiwan (KCT), the only Taiwanese kennel club recognized by the FCI; its certificates are also accepted by the UK Kennel Club and the AKC [23]. These were the only animals for which an independent breed reference existed. In six of them (Individuals 05–10), both services returned breed calls matching the pedigree. This finding demonstrates concordance among pedigree records and both commercial services for these six control dogs, but does not establish the accuracy of either service for pit bull-type dogs. The discordance observed for Individual 04 further illustrates that pedigree status alone did not guarantee concordant assignments between the two services. Concordance appeared instead to depend on whether a breed was adequately represented, and consistently labeled, in each provider’s reference panel.

Embark^®^ assigned a 100% Formosan Mountain Dog signature to the KCT-pedigreed dog (Individual 04), but also to Individual 01, and 75.4% to Individual 03 (Table 3), both of which their owners had classified visually as mixed. Orivet Genetic Pet Care returned more than ten breeds for Individuals 01 and 04, with considerable overlap between them: Japanese Shiba Inu, German Shepherd Dog, Tibetan Mastiff, Chow Chow, Anatolian Shepherd Dog, and an unassigned mixed component.

It was not possible to determine from these data which provider was closer to the true ancestry of these dogs. Embark^®^ may have over-assigned them to its Formosan Mountain Dog cluster. It is equally possible that the Embark^®^ results are correct and that the owner-reported label carries no information: free-roaming and mixed-breed dogs in Taiwan derive substantially from native stock, and Embark^®^ maintains a regional reference database for Taiwan. No independent ancestry data were available for Individuals 01 and 03.

The Orivet Genetic Pet Care panel, however, contains no Formosan Mountain Dog reference. A genotype with no corresponding cluster is distributed across whichever clusters resemble it most, and the expected outcome is a long list of breeds with no dominant contributor—which is what was obtained. Marker ascertainment may contribute as well. Commercial SNP panels have been developed largely on European and North American breeds, and their informativeness for East Asian native dogs is correspondingly uncertain; in African and Asian sheep, an apparent inability to separate populations proved to reflect limited marker informativeness rather than low genetic variability [24]. Native Taiwanese dogs also mate with little human control, as do the Korean Jindo and Poongsan, and the higher polymorphism this produces [25] leaves fewer breed-diagnostic alleles for any single cluster to capture. Neither factor need be invoked, however, to explain the result for a breed that the FCI recognized only in 2015 and that is absent from one of the two panels evaluated.

Orivet Genetic Pet Care stated that there is no American Bully breed in their database; therefore, individuals whose Embark^®^ breed signatures involved American Bully (Individuals 12, 13, and 20) appeared as American Staffordshire Terrier, or as combinations of American Staffordshire Terrier and bulldogs, in the Orivet Genetic Pet Care results. This is expected, as the American Bully is among the most recent breeds and is recognized only by kennel clubs in the United States among the pit bull-type dogs. Because the American Bully is not listed in the Taiwanese declaration, this database gap has direct regulatory consequences, which are considered below.

The clearest pattern in this study involved the American Pit Bull Terrier and the American Staffordshire Terrier. Orivet Genetic Pet Care did not report American Pit Bull Terrier in any of the 21 dogs. In Individuals 18 and 19, where Embark^®^ reported American Pit Bull Terrier at 100% and 50%, respectively, Orivet Genetic Pet Care reported American Staffordshire Terrier at exactly the same percentages. Similarly, Individual 02 was reported by Embark^®^ as 83.7% American Pit Bull Terrier and 16.3% American Staffordshire Terrier, whereas Orivet Genetic Pet Care reported this dog as 100% American Staffordshire Terrier. These results suggest that, at least for these dogs, the difference between the two platforms reflects how the two closely related breeds are assigned rather than a major difference in the estimated ancestry proportions.

A similar pattern was seen with Embark^®^. American Staffordshire Terrier was reported in only one dog (Individual 02, 16.3%). All three dogs visually identified as American Staffordshire Terriers for which Embark^®^ results were available (Individuals 16, 17, and 18) were instead reported as 10% American Pit Bull Terrier. Thus, neither service clearly separated American Pit Bull Terrier from American Staffordshire Terrier in this sample, although the two services tended to assign the dogs to different breed names.

This finding is plausible given the history of the two breeds. They shared a common gene pool before the American Staffordshire Terrier was established as a separate registered breed by the AKC in the 1930s, while the FCI does not recognise the American Pit Bull Terrier [2,3]. Their close historical relationship may therefore make genetic separation dependent on the composition and classification of the reference populations used by each provider. Because these reference data and classification algorithms are proprietary, the basis for the different assignments observed here cannot be determined directly.

Failed tests occurred in 1/21 samples for Embark^®^ and 4/21 for Orivet Genetic Pet Care, a difference that did not reach statistical significance in this small sample (Fisher’s exact test, *p* = 0.34). The providers use different sample collection methods and processing protocols, which could potentially influence sample adequacy. Embark^®^ uses a sponge swab targeting saliva, in which up to 74% of the DNA may derive from leukocytes [26], whereas Orivet Genetic Pet Care uses a nylon swab targeting buccal epithelial cells. Saliva collected with a dedicated kit has been reported to yield DNA of higher quantity and quality than buccal swabs [27], and the stabilising fluid supplied with the Embark^®^ kit may further limit degradation; the Orivet Genetic Pet Care protocol additionally requires a 15–20 min air-drying step that is vulnerable to inconsistent execution. Because both samples from each dog were collected by the same owner on the same occasion, operator variability is at least partially controlled. These differences in sample type and kit design are therefore plausible contributors to the observed difference in failure frequency. However, because sample-specific DNA quantity, quality-control metrics, and definitive causes of failure were not provided by the companies, the reason for the observed difference in failure frequency cannot be determined from this study. Given the small number of failures, these proportions should not be interpreted as comparative performance estimates.

Agreement between the breed reported by the owner or KCT and the DNA results was low. Among the seven pit bull-type dogs for which both services returned results and a specific owner-reported breed was available, only two (Individuals 14 and 15) showed agreement across all sources. This finding is consistent with previous reports of low concordance between visual and genetic breed identification [1,6,7,8]. However, the DNA results in this study were not independently validated, and the two services identified the same primary breed in only 8 of the 16 dogs for which both returned results. Therefore, disagreement between visual and genetic identification cannot be used to determine which method was incorrect. In contrast, the six pedigreed control dogs showed complete agreement among visual identification, pedigree records, and both DNA services. Such agreement was not observed for the pit bull-type or native Taiwanese dogs.

The declaration lists the American Pit Bull Terrier and the American Staffordshire Terrier, but not the Staffordshire Bull Terrier or the American Bully. The discordances we observed therefore do not all carry the same weight.

Confusion between the American Pit Bull Terrier and the American Staffordshire Terrier, discussed above, matters little for enforcement: both breeds are listed, so an error in either direction leaves the regulatory outcome unchanged. The American Bully is a different case, and discordances occurred in both directions. The Orivet Genetic Pet Care reference panel does not contain the American Bully, and all three dogs visually identified as American Bullies for which that service returned a result (Individuals 11, 12 and 13) were assigned a majority American Staffordshire Terrier signature. These dogs would therefore be classified as belonging to a listed breed, exposing their owners to confiscation or penalty on the basis of a test result. Conversely, Embark^®^ reported an American Bully signature for two dogs whose owners identified them as American Pit Bull Terriers (Individuals 20 and 21); these dogs would fall outside the declaration entirely.

In their present form, neither service provides evidence sufficient to determine whether a given dog belongs to a listed breed. Two requirements are currently unmet: disclosure of which breeds are included in the reference panel and how closely related registry breeds are separated within it, and publication of independently validated error rates for the breeds named in the declaration. Both companies state that their products are intended for companion-animal use and not for legal or forensic purposes, and nothing here should be read as validating them for that role. Until such data are available, a negative result is more defensible than a positive one, although this assumes that the panel contains the breed in question.

The study has several limitations. Most importantly, no gold standard for breed identity was available for the pit bull-type dogs; only the six pedigreed controls had an independent reference. The results therefore describe agreement between methods; they do not estimate diagnostic accuracy. Owner-reported breed may also be biased by the regulatory context in Taiwan, where owners have an incentive to report banned pit bull-type dogs as other breeds, potentially contributing to the discordance in Individuals 16, 17, 20, and 21. Samples were collected by owners for privacy reasons, and no duplicate samples were submitted, preventing assessment of sampling consistency or within-laboratory repeatability. The sample was also small and non-random (21 dogs, including 12 pit bull-type dogs), with most controls being show dogs. Because the denominators for several comparisons were small, the reported proportions are highly sensitive to the outcome of individual dogs and should be interpreted as descriptive observations rather than population-level estimates of concordance. Finally, the providers use proprietary marker panels, reference populations, and ancestry-inference algorithms that could not be independently evaluated. Embark^®^ also does not report breed contributions below 5%, which may obscure minor pit bull-type ancestry in mixed dogs and is particularly relevant in a regulatory setting.

## 5. Conclusions

We compiled kennel-club breed standards together with the enforcement guidance issued under the United Kingdom Dangerous Dogs Act 1991 into a reference intended to support first-line visual identification of pit bull-type dogs, and we compared two commercial SNP-based breed identification services. Embark^®^ offered broader coverage of the breeds relevant to Taiwan (all four pit bull-type breeds and the Formosan Mountain Dog are represented), together with a lower failure rate and downloadable raw data, and is on these grounds the more suitable of the two for use in Taiwan. This advantage concerns coverage and auditability, not demonstrated accuracy. Substantial discordance was observed between the two services in assignments involving the American Pit Bull Terrier and American Staffordshire Terrier, and the services reported different primary breeds in half of the dogs for which both returned a result. Discordant assignments involving the American Bully could also lead to different regulatory classifications under the Taiwanese declaration. Commercial DNA breed identification, as currently offered, is therefore not yet adequate as a stand-alone basis for enforcement, and adoption of any single service would require prior validation against dogs of known ancestry, with published error rates for the listed breeds. Given that no DNA-based breed identification service is available in Taiwan, this pilot study nonetheless provides the first empirical basis for that assessment.

## Figures and Tables

**Table 1:** American Pit Bull Terrier, Staffordshire Bull Terrier, American Staffordshire Terrier, and American Bully breed standards based on the Fédération Cynologique Internationale (FCI), American Kennel Club (AKC), The Kennel Club (tKC), and United Kennel Club (UKC).

	American Pit Bull Terrier (UKC) [4]	American Staffordshire Terrier (AKC, FCI) [2,3]	Staffordshire Bull Terrier (AKC, tKC, UKC, FCI) [2,3,4,5]	American Bully (UKC) [4]
Height	Male: 18–21 in (45.7–53.3 cm); Female: 17–20 in (43.2–50.8 cm)	Male: 18–19 in (45.7–48.2 cm); Female: 17–18 in (43.2–45.7 cm)	14–16 in (35.5–40.5 cm)	Male: 17–20 in (43.2–50.8 cm); Female: 16–19 in (40.5–48.2 cm)
Weight	Male: 35–60 lb (15.9–27.2 kg); Female: 30–50 lb (13.6–22.7 kg)	Male: 55–70 lb (24.9–31.8 kg); Female: 40–55 lb (18.1–24.9 kg)	Male: 28–38 lb (12.7–17.2 kg); Female: 24–34 lb (10.9–15.4 kg)	Varies
Head	Medium length, broad blunt wedge. Muzzle broad and deep, ratio approx. 2:3. Ears small to medium, set higher than top of head. Wrinkles on forehead when concentrating.	Medium length, deep through. Muzzle of medium length, rounded upper side. Ears small and thin, rose or half-prick.	Short, deep through, broad skull. Muzzle a short foreface. Ears small and thin, rose or half-prick.	Large and broad. Muzzle broad and blocky, 25–35% of head length. Ears set high, prick or flat.
Nose	Large, with wide open nostrils. May be any color.	—	Black.	Large, with well-opened nostrils. May be any color.
Eyes	Medium size, round. All colors acceptable except blue.	Dark, round.	Round, medium size; dark preferred, may relate to coat color.	Medium size, oval to slightly round.
Chest	Should never be wider than it is deep.	—	—	—
Tail	Natural extension of the topline, tapering to a point. Low set, extending to the hock; level with the backline when moving.	Short, low set, tapering to a fine point.	Medium length; does not curl much.	Low set, extending to the hock when relaxed; level with the topline when moving.
Coat	Smooth and close. Any color except merle.	Short and close. Any color.	Red, fawn, white, black, brindle, or blue, with or without white.	Smooth and close. Any color except merle.

— not specified in the referenced breed standards.

**Table 2:** Comparison between Embark^®^ and Orivet Genetic Pet Care.

	Embark^®^	Orivet Genetic Pet Care
Institute in collaboration with	Cornell University	Not mentioned
Number of SNPs	Up to 230,000	Not mentioned
Database	400+ breeds	220 breeds
Swabbing method	Genotek PG-100 sponge swab; targets saliva (white blood cells)	Sterile nylon swab; targets buccal epithelial cells
Suggested swabbing duration	30–60 s	Approximately 10 s
Drying after swabbing	Not required	Approximately 15–20 min
Raw data	Available	Not available
Pit bull-type breeds tested	APBT, SBT, AmStaff, American Bully	APBT, SBT, AmStaff
Failed tests	1/21	4/21
Formosan Mountain Dog	In database	Not in database

APBT, American Pit Bull Terrier; SBT, Staffordshire Bull Terrier; AmStaff, American Staffordshire Terrier.

**Table 3:** DNA breed identification results of 21 dogs by Embark^®^ and Orivet Genetic Pet Care.

No.	Visually Identified Breed/Breed Approved by KCT	Embark^®^	Orivet Genetic Pet Care
01	Mixed dog	Formosan Mountain Dog (100%)	Unknown mixed breed (20.73%), Japanese Shiba Inu (13.19%), Korean Jindo (12.29%), German Shepherd (11.8%), Tibetan Mastiff (8.1%), Manchester Terrier (7.75%), Japanese Spitz (5.51%), Whippet (4.85%), Chow Chow (4.31%), Anatolian Shepherd Dog (4.03%), etc.
02	Pit bull-type dog	American Pit Bull Terrier (83.7%), American Staffordshire Terrier (16.3%)	American Staffordshire Terrier (100%)
03	Mixed dog	Formosan Mountain Dog (75.4%), German Shepherd (24.6%)	Typing failed
04	Formosan Mountain Dog	Formosan Mountain Dog (100%)	Unknown mixed breed (22.49%), Tibetan Mastiff (17.51%), German Shepherd (12.62%), Shar Pei (12.04%), Chihuahua (7.58%), etc.
05	German Shepherd	German Shepherd (100%)	German Shepherd (100%)
06	Labrador Retriever	Labrador Retriever (100%)	Labrador Retriever (100%)
07	Golden Retriever	Golden Retriever (100%)	Golden Retriever (100%)
08	French Bulldog	French Bulldog (100%)	French Bulldog (100%)
09	English Bulldog	Bulldog (100%)	English Bulldog (100%)
10	Boxer	Boxer (100%)	Boxer (100%)
11	American Bully	Typing failed	American Staffordshire Terrier (52.08%), British Bulldog (34.97%), French Bulldog (12.95%)
12	American Bully	American Bully (75.4%), English Bulldog (24.6%)	American Staffordshire Terrier (52.08%), British Bulldog (34.97%), French Bulldog (12.95%)
13	American Bully	American Bully (100%)	American Staffordshire Terrier (87.5%), English Bulldog (12.5%)
14	Staffordshire Bull Terrier	Staffordshire Bull Terrier (100%)	Staffordshire Bull Terrier (100%)
15	Staffordshire Bull Terrier	Staffordshire Bull Terrier (100%)	Staffordshire Bull Terrier (100%)
16	American Staffordshire Terrier	American Pit Bull Terrier (100%)	Typing failed
17	American Staffordshire Terrier	American Pit Bull Terrier (100%)	Typing failed
18	American Staffordshire Terrier	American Pit Bull Terrier (100%)	American Staffordshire Terrier (100%)
19	American Pit Bull Terrier	American Pit Bull Terrier (50%), Neapolitan Mastiff (50%)	American Staffordshire Terrier (50%), Neapolitan Mastiff (50%)
20	American Pit Bull Terrier	American Bully (81.6%), English Mastiff (18.4%)	American Staffordshire Terrier (100%)
21	American Pit Bull Terrier	American Bully (100%)	Typing failed

KCT, Kennel Club of Taiwan. Percentages indicate the proportion of each breed within the reported breed signature.

## Data Availability

The data presented in this study are available from the corresponding author upon reasonable request.
